# Through‐Knee Amputation Following Total Knee Arthroplasty With Retention of Femoral Component: A Case Report

**DOI:** 10.1155/cro/4486143

**Published:** 2026-07-14

**Authors:** Katie Hutchinson, Alex Trompeter

**Affiliations:** ^1^ Department of Trauma and Orthopaedics, St George′s University Hospitals NHS Foundation Trust, London, UK; ^2^ St George′s Medical School, City St George′s University of London, London, UK

**Keywords:** extensor mechanism failure, femoral component retention, limb salvage, through-knee amputation, total knee arthroplasty

## Abstract

**Introduction:**

Extensor mechanism disruption following total knee arthroplasty (TKA) is an uncommon but serious complication. Surgical management is often complex, particularly in cases of failed reconstruction or persistent instability, and may ultimately necessitate amputation.

**Case Presentation:**

We report a 79‐year‐old female with a history of TKA and prior patellar tendon repair, who developed a fixed flexion deformity and periprosthetic knee dislocation. Given the failure of conventional interventions and compromised soft tissues, she underwent a through‐knee amputation. The femoral component of the prosthesis was deliberately retained, a strategy that, to our knowledge, has not previously been described in the literature. Postoperatively, the patient experienced significant pain relief, and the residual limb was suitable for prosthetic fitting, although she elected for wheelchair‐based mobility.

**Discussion:**

Retention of the femoral component during through‐knee amputation presents unique surgical challenges but may offer functional and biomechanical advantages, including a stable, end‐bearing residual limb and preservation of limb length. This case highlights the feasibility, rationale, and potential benefits of femoral component retention in the context of complex post‐TKA limb salvage.

**Conclusion:**

Through‐knee amputation with retention of the femoral component represents a novel salvage approach in selected patients with failed TKA reconstruction, providing both anatomical and functional advantages while broadening the management strategies for complex knee arthroplasty complications.

## 1. Introduction

Extensor mechanism disruption following total knee arthroplasty (TKA) is a rare but debilitating complication, with a reported incidence ranging from 0.1% to 2.5% [1–4]. Risk factors include prior knee surgery, infection and trauma [1, 4]. Clinically, extensor mechanism failure, such as patellar tendon rupture or patellar fracture, can result in loss of active knee extension, extensor lag and impaired ambulation [5]. Functional impairment is often profound, and even with intervention, many patients fail to regain preinjury mobility.

Accurate diagnosis requires a detailed understanding of knee anatomy, with careful differentiation between the quadriceps tendon, the patellar tendon and the patellar disruptions. Imaging modalities, including plain radiographs, ultrasound or MRI, are often necessary to assess the integrity of both the extensor mechanism and the prosthesis.

Management strategies depend on the timing and severity of disruption. Nonsurgical measures, including bracing and walking aids, are typically reserved for patients with low functional demand or high surgical risk but often provide limited improvement. Surgical options include primary repair, autograft or allograft reconstruction, with variable success rates and substantial risk of complications such as infection, rerupture and persistent extensor lag [6]. Early acute ruptures may be amenable to direct repair, whereas chronic or late disruptions usually require complex reconstruction.

Amputation becomes a consideration in cases of failed reconstruction, persistent periprosthetic infection or severe functional compromise. Knee disarticulation, also known as through‐knee amputation, may be preferable to transfemoral amputation in some instances, offering advantages such as improved prosthetic end‐bearing, preservation of limb length and a reduced risk of hip contracture [7, 8]. Patient comorbidities, ambulatory potential and local tissue viability should guide decision‐making.

We present the case of a 79‐year‐old female with a history of TKA and subsequent patellar tendon repair, who developed a fixed flexion deformity and periprosthetic knee dislocation. Given the failure of prior interventions and compromised extensor mechanism, she underwent a through‐knee amputation with retention of the femoral component. To our knowledge, this approach has not been previously reported in the literature, and this case highlights the clinical challenges, surgical considerations and potential functional outcomes associated with this rare salvage procedure.

## 2. Case Report

**Figure 1:**
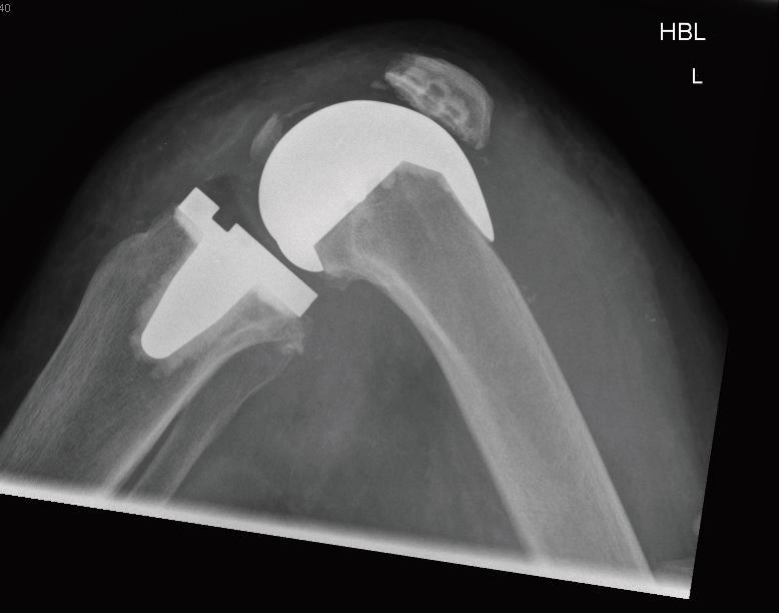
Lateral x‐ray of left knee, showing TKR in situ with failed extensor mechanism.

A 79‐year‐old female with a background of left TKA 3 years prior and patellar tendon repair 10 weeks earlier presented in November 2018 (Figure 1) with an acute onset of pain, swelling and deformity of the left knee. On examination, the knee was warm to palpation, grossly swollen and held in full flexion. The patient was unable to extend the knee due to severe pain.

Radiographs demonstrated a failed extensor mechanism. The patient was managed conservatively in a cricket pad splint and discharged after a period of inpatient care.

In January 2019, 2 weeks following discharge, she re‐presented with worsening pain and functional decline. She reported that since returning home, she had been unable to mobilise and had relied on family for assistance. Examination revealed a fixed flexion deformity of the left knee with a restricted range of motion (90°–100°), inability to extend the knee and loss of straight‐leg raise.

**Figure 2:**
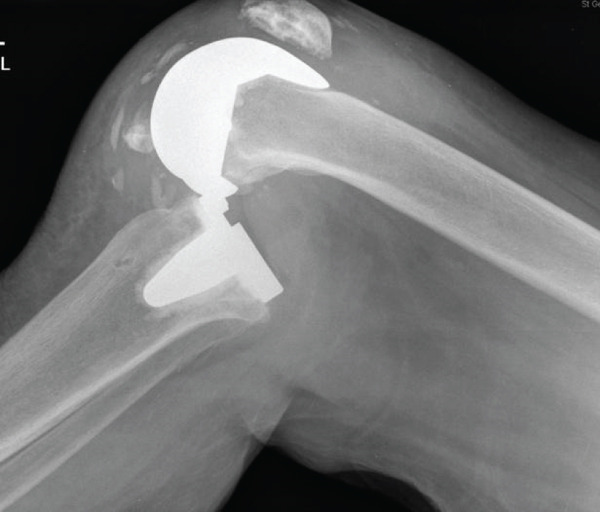
Lateral XR view of left knee showing posterior dislocation of tibial end of TKR.

**Figure 3:**
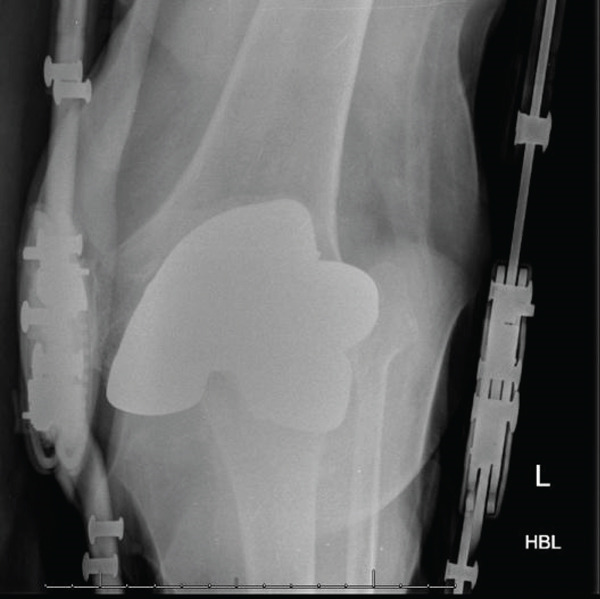
Anterior–posterior XR view of the left knee showing posterior dislocation of the tibial end of TKR.

**Figure 4:**
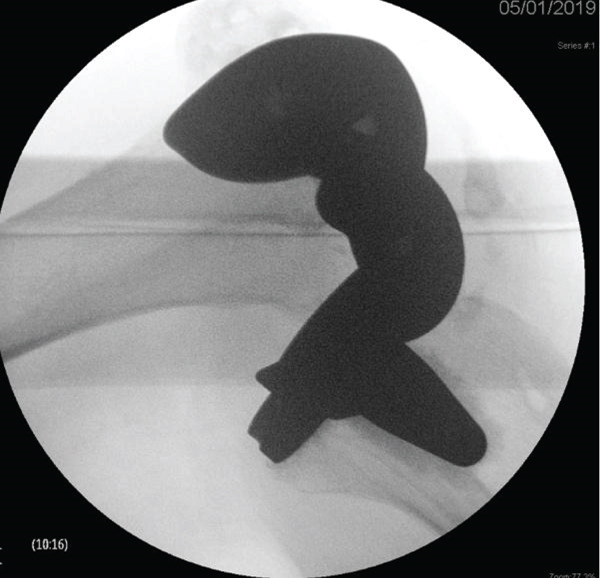
Lateral XR of failed reduction under anaesthesia of left TKR intraoperatively.

**Figure 5:**
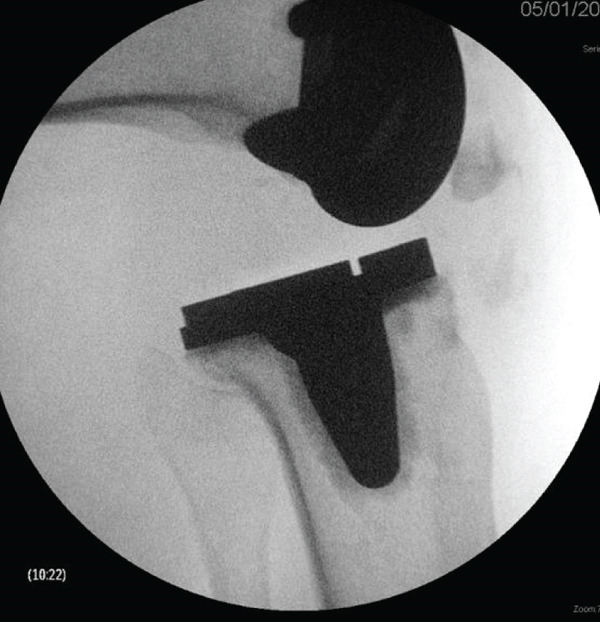
Intraoperative XR of failed reduction under anaesthesia of left TKR.

X‐rays demonstrated a periprosthetic knee dislocation (Figures 2 and 3). Closed reduction in the emergency department was unsuccessful, and examination under anaesthesia the following day also failed (Figures 4 and 5). Given the persistent extensor mechanism deficiency, fixed flexion deformity and failed attempts at reduction, a knee arthrodesis was initially considered.

**Figure 6:**
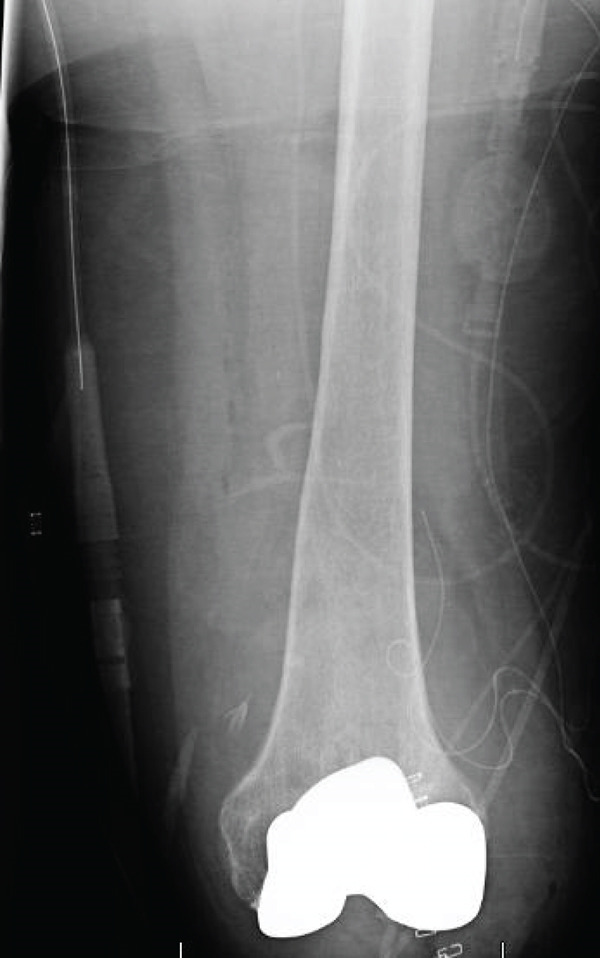
Anterior posterior x‐ray view showing through‐knee amputation of left leg, with retained femoral component of TKR.

**Figure 7:**
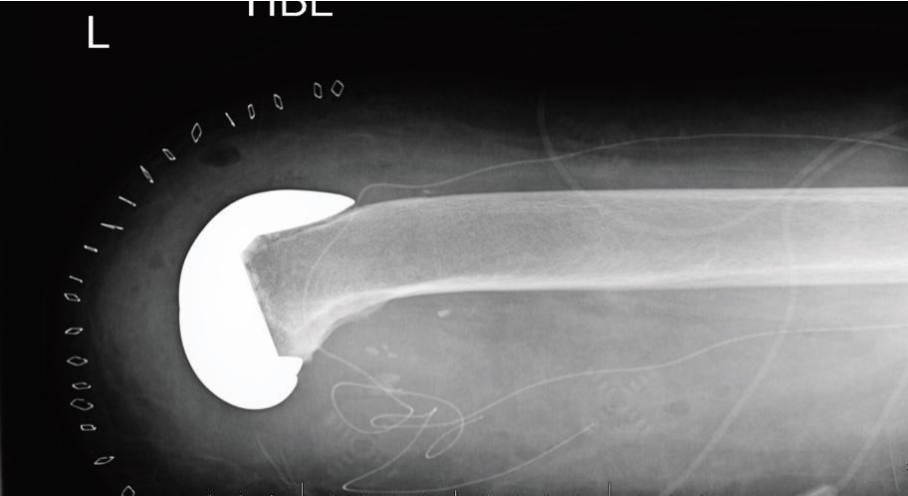
Lateral x‐ray view showing through‐knee amputation of left leg, with retained femoral component of TKR.

In November 2019, following continued deterioration and poor functional outcome, the patient underwent a left through‐knee amputation under general anaesthesia with femoral nerve block. The patella was excised, and the femoral component of the TKA was found to be well‐fixed and therefore retained in situ (Figures 6 and 7). Dissection proceeded with ligation of the tibial artery and vein, division of the tibial and common peroneal nerves and isolation of the gastrocnemius, which was used for myodesis to the quadriceps. The wound was closed in layers using 3‐0 Monocryl and skin staples, with standard dressing applied. A standard postoperative regimen was implemented, consisting of two doses of prophylactic antibiotics and the administration of dalteparin.

**Figure 8:**
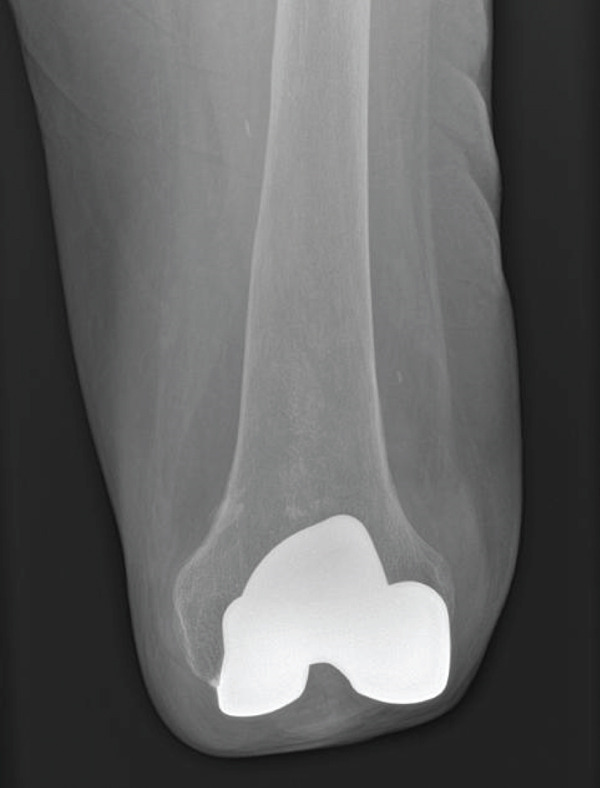
6 year follow‐up of through‐knee amputation with retained femoral component of TKR.

**Figure 9:**
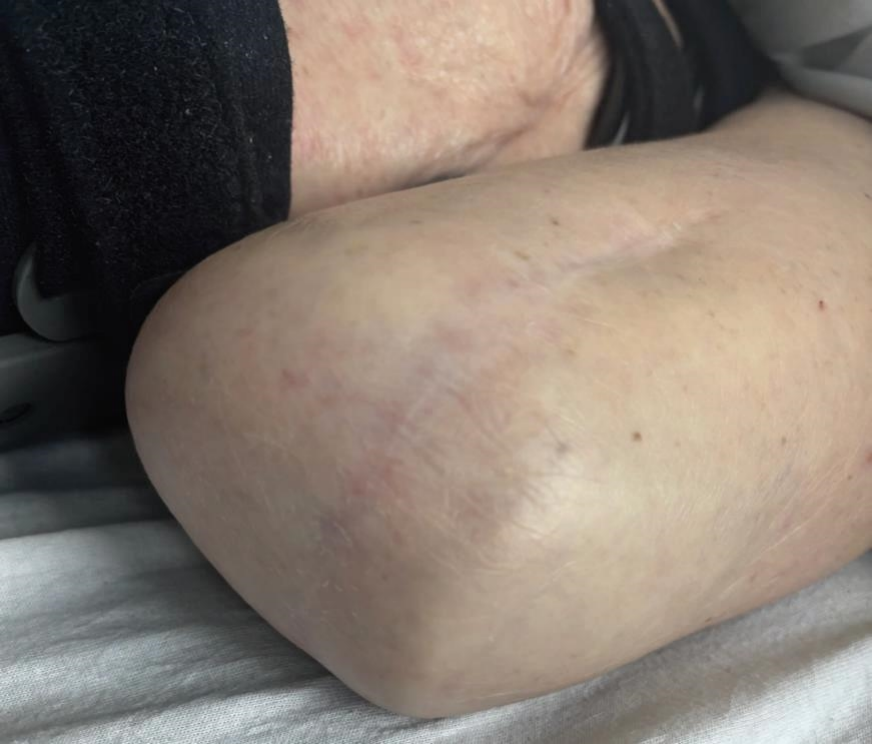
Healed stump 6 years after through‐knee amputation.

Following the amputation, the patient reported a marked improvement in pain compared with her preoperative state. She was assessed and deemed eligible for a prosthesis fitting, given the satisfactory residual limb contour and preserved end‐bearing potential from the through‐knee level (Figures 8 and 9). However, she declined prosthetic rehabilitation, opting instead for wheelchair‐based mobility.

The case presented was further compounded by the prolonged period of deterioration before definitive intervention. Multiple failed reduction attempts and nearly a year of conservative management, despite persistent instability, declining function and worsening pain, highlight the difficulty of achieving meaningful limb salvage in this context. The rationale for the extended nonoperative period included serial assessments of soft‐tissue viability, attempts to optimise mobility through physiotherapy, and consideration of reconstructive options, such as knee arthrodesis. However, the continued medical deterioration ultimately necessitated reconsideration of the treatment plan.

## 3. Discussion

Performing a through‐knee amputation, or knee disarticulation, in a patient with a total knee replacement prosthesis in situ presents several unique surgical and postoperative challenges. The presence of a well‐fixed femoral component necessitates meticulous dissection to preserve the integrity of the implant while ensuring adequate soft tissue coverage and vascular control. Retention of the femoral component may complicate flap design, as conventional myodesis and muscle coverage techniques often require modification to accommodate the prosthesis and to achieve a stable, end‐bearing residual limb. Furthermore, the risk of infection is markedly increased, particularly in the context of prior arthroplasty, recent surgical intervention or compromised soft tissue viability, necessitating stringent aseptic technique and appropriate perioperative antibiotic prophylaxis. Neurovascular structures, including the tibial and common peroneal nerves, must be handled with precision to minimise neuroma formation and postoperative pain. Additionally, consideration must be given to bone stock, alignment and prosthetic compatibility, as the retained femoral component may impact residual limb length, socket design and long‐term functional outcomes.

Arthrodesis was considered as a limb salvage option; however, following multidisciplinary discussion involving the patient, their family and the treating teams, a decision was made to proceed with amputation in accordance with patient preference and overall clinical context. Within our institution, through‐knee disarticulation is a well‐established procedure with extensive experience in prosthetic rehabilitation and is not considered a barrier to functional recovery, including in patients with a primary goal of independent mobility with prosthetic use.

Concerns regarding prosthetic‐related complications, including stump pressure, skin breakdown and potential issues related to load transmission at the prosthetic interface, were considered during decision making. These risks were balanced against expected functional outcomes and the anticipated rehabilitation trajectory.

Retaining the femoral component can confer biomechanical advantages by providing a stable, end‐bearing stump suitable for prosthetic rehabilitation [1, 4, 9]. The through‐knee amputation offers distinct functional benefits compared with above‐knee amputation, including a longer lever arm, improved muscle control and a reduced risk of hip flexion contracture. In patients with prior TKA, retention of a well‐fixed femoral component during knee disarticulation can further enhance residual limb function but requires careful surgical planning to address soft tissue coverage, vascular control and prosthetic alignment. At our centre, through‐knee amputations have been associated with favourable rehabilitation outcomes and are often considered functionally advantageous compared with transfemoral amputations, particularly with regard to sitting balance and end‐bearing potential. The level of amputation is determined on a case‐by‐case basis through MDT discussion involving orthopaedic surgeons, rehabilitation physicians and prosthetists, with decisions guided by expected postoperative functionality and patient preference.

Comparative studies and prosthetist surveys have demonstrated that through‐knee amputation yields similar or superior rates of wound healing, ambulation and independent living compared with above knee amputation, with some series reporting up to 70% successful ambulation and satisfactory functional outcomes even among high‐risk vascular patients [2, 4, 9].

Patient‐reported outcomes following through‐knee and above‐knee amputations are generally similar in terms of pain, prosthesis use, satisfaction and quality of life. However, a proportion of patients may remain wheelchair‐dependent or experience chronic residual limb pain regardless of level [10] [11, 12]. Although prosthetists often favour through‐knee amputation for its end‐bearing capability and mechanical stability, above‐knee amputation is sometimes preferred for cosmetic reasons and greater flexibility in prosthetic component selection. In patients pursuing prosthetic ambulation after retention of a femoral component, potential concerns include increased socket pressure over the implant, skin compromise and theoretical risk of prosthesis‐related loosening; however, evidence regarding these risks remains limited.

There remains a significant evidence gap, as no high‐quality comparative or randomised controlled studies have yet defined the superiority of through‐knee amputation with a retained femoral component over above‐knee amputation in terms of function, rehabilitation or complication rates [7, 13] [12, 14]. Further research is required to clarify the long‐term outcomes of prosthesis retention in this context and to establish best practice for these rare, complex cases.

## 4. Conclusion

In summary, performing a through‐knee amputation with retention of the femoral component of a total knee replacement prosthesis may provide several theoretical advantages, including a stable, end‐bearing stump, improved muscular control and reduced risk of hip contracture. These benefits may translate into comparable or superior wound healing and ambulation outcomes relative to above‐knee amputation. However, functional performance, patient satisfaction and complication rates appear broadly similar between the two approaches, and the choice of amputation level should therefore be individualised based on patient comorbidities, soft tissue condition and surgical feasibility.

## Funding

No funding was received for this manuscript.

## Consent

No written consent has been obtained from the patients, as no patient‐identifiable data were included in this case report.

## Conflicts of Interest

Katie Hutchinson declares no conflicts of interest. Alex Trompeter: Consultancy (research and development): Stryker Trauma, Orthosolutions/Meshworks Orthofix, Medical Insights; Consultancy (education): Stryker Smith & Nephew Orthofix Orthosolutions/Meshworks; Royalties: Stryker Trauma, Oxford University Press, JP Medical Publishing; Professional and editorial roles: member, BOA Clinical Standards Committee Member, BOA Trauma Committee Research Chair and Executive Committee Member, BLRS Editorial Board Member, Bone & Joint Journal (BJJ) Deputy Editor, European Journal of Orthopaedic Surgery & Traumatology (EJOST).

## Data Availability

The data that support the findings of this study are available on request from the corresponding author. The data are not publicly available due to privacy or ethical restrictions.
